# Endothelial dysfunction and altered endothelial biomarkers in patients with post-COVID-19 syndrome and chronic fatigue syndrome (ME/CFS)

**DOI:** 10.1186/s12967-022-03346-2

**Published:** 2022-03-22

**Authors:** Milan Haffke, Helma Freitag, Gordon Rudolf, Martina Seifert, Wolfram Doehner, Nadja Scherbakov, Leif Hanitsch, Kirsten Wittke, Sandra Bauer, Frank Konietschke, Friedemann Paul, Judith Bellmann-Strobl, Claudia Kedor, Carmen Scheibenbogen, Franziska Sotzny

**Affiliations:** 1 Institute for Medical Immunology, Charité–Universitätsmedizin Berlin, Corporate Member of Freie Universität Berlin and Humboldt Universität Zu Berlin, Berlin, Germany; 2 Berlin Institute of Health at Charité–Universitätsmedizin Berlin, BIH Center for Regenerative Therapies (BCRT), Berlin, Germany; 3 DZHK (German Center for Cardiovascular Research), Partner Site Berlin, Berlin, Germany; 4 Department of Cardiology, Charité–Universitätsmedizin Berlin, Corporate Member of Freie Universität Berlin and Humboldt Universität Zu Berlin, Berlin, Germany; 5 Center for Stroke Research Berlin (CSB), Charité–Universitätsmedizin Berlin, Corporate Member of Freie Universität Berlin and Humboldt Universität Zu Berlin, Berlin, Germany; 6 Institute of Biometry and Clinical Epidemiology, Charité–Universitätsmedizin Berlin, Corporate member of Freie Universität Berlin and Humboldt Universität Zu Berlin, Berlin, Germany; 7 Experimental and Clinical Research Center (ECRC), Charité–Universitätsmedizin Berlin, Corporate Member of Freie Universität Berlin and Humboldt Universität Zu Berlin, Berlin, Germany; 8 NeuroCure Clinical Research Center (NCRC), Charité–Universitätsmedizin Berlin, Corporate Member of Freie Universität Berlin and Humboldt Universität Zu Berlin, Berlin, Germany; 9 Max Delbrueck Center for Molecular Medicine, Experimental and Clinical Research Center (ECRC), Berlin, Germany

**Keywords:** Post-COVID syndrome, Myalgic encephalomyelitis/chronic fatigue syndrome, Endothelial dysfunction, Reactive hyperaemia index, Endothelin-1

## Abstract

**Background:**

Fatigue, exertion intolerance and post-exertional malaise are among the most frequent symptoms of Post-COVID Syndrome (PCS), with a subset of patients fulfilling criteria for Myalgic Encephalomyelitis/Chronic Fatigue Syndrome (ME/CFS). As SARS-CoV-2 infects endothelial cells, causing endotheliitis and damaging the endothelium, we investigated endothelial dysfunction (ED) and endothelial biomarkers in patients with PCS.

**Methods:**

We studied the endothelial function in 30 PCS patients with persistent fatigue and exertion intolerance as well as in 15 age- and sex matched seronegative healthy controls (HCs). 14 patients fulfilled the diagnostic criteria for ME/CFS. The other patients were considered to have PCS. Peripheral endothelial function was assessed by the reactive hyperaemia index (RHI) using peripheral arterial tonometry (PAT) in patients and HCs. In a larger cohort of patients and HCs, including post-COVID reconvalescents (PCHCs), Endothelin-1 (ET-1), Angiopoietin-2 (Ang-2), Endocan (ESM-1), IL-8, Angiotensin-Converting Enzyme (ACE) and ACE2 were analysed as endothelial biomarkers.

**Results:**

Five of the 14 post-COVID ME/CFS patients and five of the 16 PCS patients showed ED defined by a diminished RHI (< 1.67), but none of HCs exhibited this finding. A paradoxical positive correlation of RHI with age, blood pressure and BMI was found in PCS but not ME/CFS patients. The ET-1 concentration was significantly elevated in both ME/CFS and PCS patients compared to HCs and PCHCs. The serum Ang-2 concentration was lower in both PCS patients and PCHCs compared to HCs.

**Conclusion:**

A subset of PCS patients display evidence for ED shown by a diminished RHI and altered endothelial biomarkers. Different associations of the RHI with clinical parameters as well as varying biomarker profiles may suggest distinct pathomechanisms among patient subgroups.

**Supplementary Information:**

The online version contains supplementary material available at 10.1186/s12967-022-03346-2.

## Background

Persistent symptoms for more than six months following mild to moderate coronavirus disease-2019 (COVID-19) are reported in 10–30% of patients [1–3]. The WHO recently defined the post-COVID-19 condition as a state persisting at least three months from the onset of COVID-19 with common symptoms such as fatigue, post-exertional malaise and cognitive dysfunction impacting everyday functioning.

In our observational longitudinal PA-COVID Fatigue study of PCS patients with persistent moderate to severe fatigue and exertion intolerance for more than six months after mild to moderate COVID-19, we found that approximately 50% of patients fulfilled the diagnostic criteria of Myalgic Encephalomyelitis/Chronic Fatigue Syndrome (ME/CFS), but many PCS patients not fulfilling these criteria were equally impaired [4]. ME/CFS is a complex disease frequently triggered by an infection with Epstein-Barr virus (EBV) or parvovirus B19, but several other viral and nonviral triggers have been described [5–7]. Postexertional malaise (PEM), which describes a disproportional aggravation of symptoms typically lasting for more than 14 h up to several days following a mild mental or physical exertion is a key symptom of ME/CFS [8].

The pathomechanism of ME/CFS is not well understood, but there is ample evidence for impaired perfusion and endothelial dysfunction (ED) [7, 9–12]. ED is characterised by a diminished bioavailability of vasodilators, while on the other hand, endothelium-derived vasoconstrictors are increased, leading to impaired endothelium-dependent vasodilation [13, 14]. In the context of infection with SARS-CoV-2, the potential role of ED is of particular interest, as the virus can directly infect the endothelium by engaging the Angiotensin-Converting Enzyme (ACE) 2 receptor [15]. In acute COVID-19, there is evidence for vascular endothelial cell infection, endotheliitis and microthrombosis across multiple vascularized tissues [16, 17].

In this study, we aimed to characterise peripheral endothelial function using postocclusive reactive hyperaemia peripheral arterial tonometry (RH-PAT) in PCS patients following mild to moderate COVID-19. In addition, we analysed several endothelial biomarkers including Endothelin-1 (ET-1), Angiopoietin-2 (Ang-2) and Endocan (ESM-1) which play an important role in inflammatory and noninflammatory diseases associated with ED [18–22]. Furthermore, we assessed Interleukin 8 (IL-8), Angiotensin-Converting Enzyme (ACE) and ACE2. IL-8 is secreted not only by monocytes/macrophages but also by endothelial cells and vascular smooth muscle cells. This chemokine plays an important role in (endothelial) inflammation and regulation of leukocyte rolling as well as vascular permeability [23]. ACE and ACE2 are crucial actors in the maintenance of blood pressure and vascular homeostasis [2]. ACE converts angiotensin I into the vasoconstrictive angiotensin II, which activates angiotensin II receptors. Counterregulatory ACE2 cleaves angiotensin I into angiotensin 1–9 and metabolises angiotensin II to angiotensin 1–7. Peptides generated by ACE2 are ligands of the receptor Mas, which triggers protective, vasodilative signalling [24].

## Methods

### Participants

Thirty PCS patients with persistent fatigue and exertion intolerance following mild to moderate COVID-19 were recruited from an ongoing observational study for the measurement of endothelial function by RH-PAT. Fourteen of the patients fulfilled the diagnostic criteria for ME/CFS according to the 2003 Canadian Consensus Criteria [4, 25]. In contrast to the original classification and in accordance with the studies of L. Jason and colleagues, a minimum of 14 h (instead of 24 h) of PEM was required for the diagnosis of ME/CFS [26]. As a control group, 15 age- and sex-matched HCs without a known history of COVID-19 were characterised using RH-PAT. The endothelial biomarkers were validated in a second cohort of 56 PCS patients (26 of them fulfilling diagnostic criteria for ME/CFS), 50 HCs without a known history of COVID-19, and 20 PCHCs with at least five months elapsed following COVID-19 infection. HCs of both cohorts were recruited before SARS-COV-2 vaccination and were negative for SARS-CoV-2 antibodies tested by Anti-SARS-CoV-2-spike IgG-ELISA (Euroimmune). The characteristics of both cohorts are provided in Table 1A and B. The study was approved by the Ethics Committee of Charité—Universitätsmedizin Berlin in accordance with the 1964 Declaration of Helsinki and its later amendments (EA2/066/20). All study participants gave written informed consent.

**Table 1:** Characteristics of the study groups

(A) PAT study group	ME/CFS (*n* = 14)	PCS (*n* = 16)	HC (*n* = 15)	*p* value*
Age, median (range) [years]	44.5 (24–59)	42 (27–66)	43 (23–58)	0.9491
Female sex, *n* (%)	12 (86)	15 (94)	13 (87)	0.7539
Months after COVID infection, median (range)	9 (8–11)	9 (4–12)	NA	0.3602
Heart rate, median (range) [bpm]	68.5 (51–78)	69.5 (53–89)	69 (46–84)	0.7381
Systolic blood pressure, median (range) [mmHg]	120 (99–148)	124.5 (100–169)	126 (105–147)	0.6693
Diastolic blood pressure, median (range) [mmHg]	88.5 (64–106)	87.5 (64–114)	91 (77–116)	0.7095
Body Mass Index (BMI), median (range)	23.78 (20.24–31.83	23.64 (19.36–32.18)	NA	0.6116
PEM score median (range)	33.5 (17–46)	22 (16–34)	NA	**0.0065**
Chalder Fatigue Scale median (range)	26.5 (20–33)	25.5 (15–32)	NA	0.4509
Bell Disability Scale median (range)	40 (30–80)	50 (40–80)	NA	0.3123

(B) Validation study group	ME/CFS (*n* = 26)	PCS (*n* = 30)	HC (*n* = 50)	PCHC (*n* = 20)	*p* value*
Age, median (range) [years]	46.5 (21–62)	37 (20–62)	38 (19–65)	36.5 (23–56)	0.1650
Female sex, *n* (%)	23 (88)	21 (68)	35 (70)	14 (70)	0.2696
Months after COVID infection, median (range)	8 (6–16)	8 (6–15)	NA	6 (5–8)	** < 0.0001**
PEM score median (range)	36 (20–46)	24.5 (1–42)	NA	NA	** < 0.0001**
Chalder Fatigue Scale median (range)	29 (18–33)	24 (15–32)	NA	NA	**0.0003**
Bell Disability Scale median (range)	30 (20–60)	50 (30–90)	NA	NA	**0.0010**

*For statistical analysis the Kruskal–Wallis test was used when comparing more than two groups, and the Mann–Whitney U rank-sum test was used when comparing two groups. The chi-square test was used to compare the sex distribution. A p value ≤ 0.05 was considered statistically significant

*NA* not assessed, *PAT*peripheral arterial tonometry

### Assessment of endothelial function

Peripheral endothelial function was assessed using postocclusive reactive hyperaemia peripheral arterial tonometry (RH-PAT) (endoPAT2000 device; Itamar Medical Ltd.; Caesarea, Israel) as previously described [10]. Endothelium-mediated changes in peripheral arterial tone were recorded using plethysmographic probes on the index finger of each hand during reactive hyperaemia. Hyperaemia was induced by occlusion of the left brachial artery over 5 minutes using an inflatable blood pressure cuff. The RHI was calculated from the change in the pulse wave amplitude (PWA) relative to baseline in the occluded arm and was corrected for corresponding changes in PWA relative to baseline in the contra-lateral, nonoccluded arm in order to minimise the influence of nonendothelial dependent systemic effects, using the equation: RHI = (A/B) ÷ (C/D). Based on previous studies and the manufacturer’s analytical instructions, ED was defined as RHI ≤ 1.67 [27–30]. The automatic batch analysis of individual measurements was performed using the manufacturer’s analysis version 3.1.2 (2.0).

Measurements of peripheral endothelial function were performed under standardised conditions between 8:30 am and 12:30 pm after 15 min of supine rest in a quiet, dimly lit, air-conditioned room ensuring temperatures of 21–24 °C. Blood pressure was measured using a digital blood pressure monitor on the right upper arm in order to provide orientation for the individual supra-systolic pressure of 60 mmHg that was necessary for sufficient occlusion in the later procedure. The average heart rate was determined during the five-minute preocclusion period by the endoPAT2000 device. Venous blood samples were collected 10 min following the assessment of endothelial function from the participants’ control arm where no occlusion was performed.

### Assessment of biomarkers

The serum ESM-1 concentration was measured by ELISA kits purchased from Aviscera Bioscience, Inc., serum Ang-2 was measured by Quantikine Human Angiopoietin-2 Immunoassay kits purchased from R&D Systems, Inc., and the serum ET-1 concentration was determined by QuantiGlo™ ELISA kits purchased from R&D Systems, Inc., according to the manufacturers' protocols. ACE and IL-8 (post erythrocyte lysis [31]) were determined at the Charité diagnostics laboratory (Labor Berlin GmbH, Berlin, Germany). Serum ACE2 levels were determined by ELISA kits purchased from R&D Systems.

Biomarkers were assessed in the RH-PAT study participants (Table 1A). In addition, the biomarker analysis was validated in a second cohort including post-COVID ME/CFS, PCS, HC and PCHC subjects (Table 1B).

### Assessment of symptom severity

As part of their initial consultation at the Institute of Medical Immunology at Charité Berlin, all patients completed the modified Symptom Questionnaire for PEM (DSQ-PEM) [26], the Chalder Fatigue Scale [32] and the Bell Disability Scale [33]. The DSQ-PEM uses five different 5-point Likert scales each to assess the frequency or severity of PEM symptoms and one 7-point Likert scale to assess the duration of PEM [26] with a resulting maximum score of 46. The Chalder Fatigue Scale uses eleven different 4-point Likert scales to measure the severity of fatigue [32] with a resulting maximum score of 33. The Bell Disability Scale comprises eleven statements regarding the level of physical function. The scale is scored from 0 (very severe, bedridden constantly) – 100 (healthy) in steps of ten [33].

### Statistical analysis

For comparative analysis, Kruskal–Wallis test with Dunn´s post-hoc multiple comparisons or the Mann–Whitney-U rank-sum-test for quantitative parameters were used. The ED distribution was analysed in a 2 × 2 contingency table and tested for significance by Fisher´s exact test. Correlation analysis was performed using the nonparametric Spearman coefficient. Statistical tests were performed using GraphPad Prism software (Version 6.07). A two-tailed *p* value ≤ 0.05 was considered statistically significant.

## Results

### Study population

The characteristics of the participants in the PAT study group are provided in Table 1A. Fatigue assessed by the Chalder Fatigue Scale and severity of the patient's disability assessed by the Bell Disability Scale were comparable between the two patient groups. The PEM score was higher in ME/CFS patients (median of 33.5) than in PCS patients (median 22.0). The patient groups did not differ in heart rate, blood pressure or BMI. The median time interval since COVID-19 infection was nine months in both patient groups. Endothelial biomarkers were validated in a second study cohort. The characteristics of the validation cohort are provided in Table 1B. Here, the PEM score again was higher in ME/CFS patients (median of 36) than in PCS patients (median 24.5). Fatigue assessed by the Chalder Fatigue Scale was more severe in ME/CFS patients (median of 29) than in PCS patients (median of 24), and physical function assessed by the Bell Disability Scale was worse in ME/CFS patients (median of 30) than in PCS patients (median of 50). The median time interval since COVID-19 infection was eight months in patients and six months in PCHCs.

### Evidence for peripheral ED in patients

**Fig. 1:**
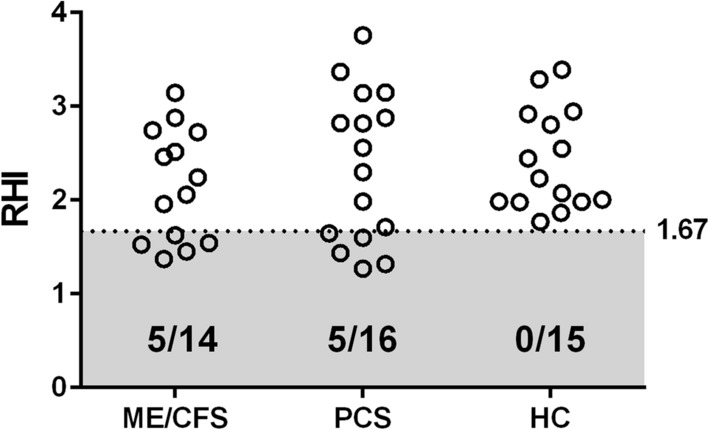
Endothelial dysfunction (ED) assessed by RH-PAT. ED was found in five of 14 ME/CFS patients and in five of 16 PCS patients but not in healthy controls (HCs). The RHI value for each patient is plotted. The dotted line indicates the RHI cut-off value of ≤ 1.67 defining ED. [RHI = reactive hyperaemia index; RH-PAT = reactive hyperaemia peripheral arterial tonometry]

Five of 14 patients with post-COVID-19 ME/CFS and five of 16 patients with PCS had peripheral ED defined by a diminished reactive hyperaemia index (RHI) (≤ 1.67), but none of the HCs exhibited this finding (Fig. 1). Patient cohorts showed a significantly higher frequency of ED than the HC group (Fisher's exact test; p_ME/CFS_ = 0.0169 and p_PCS_ = 0.0434).

### Paradoxical associations of clinical parameters with the RHI

**Fig. 2:**
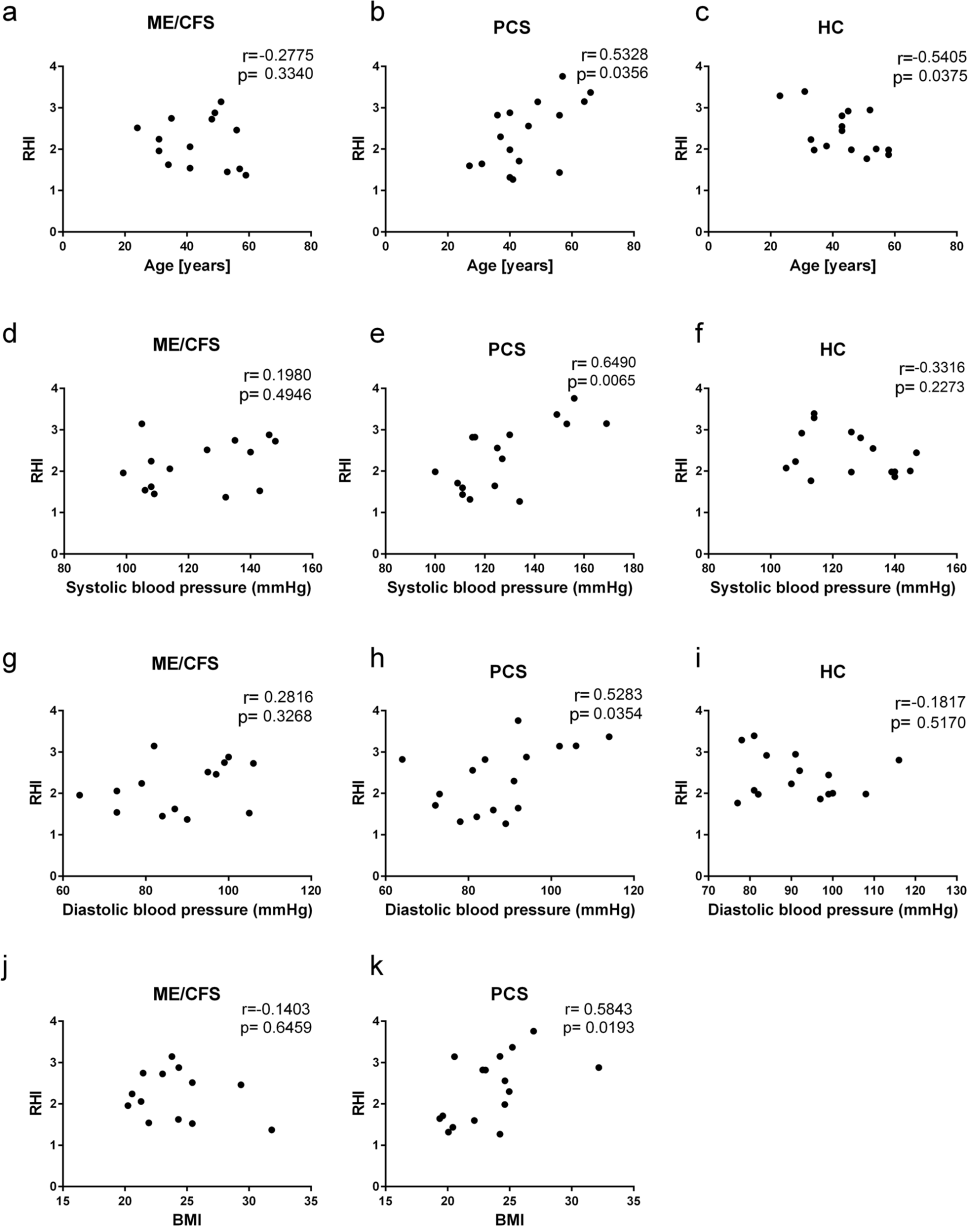
Correlation of age, blood pressure and BMI with the reactive hyperaemia index (RHI). Correlations of the RHI with age (**a–c**), systolic blood pressure (**d–f**), diastolic blood pressure (**g–i**) and BMI (**j**, **k**) for ME/CFS patients (n = 14; **a**, **d**, **g**, **j**), PCS patients (n = 16; **b**, **e**, **h**, **k**) and healthy controls (HC) (n = 15; **c**, **f**, **i**). Correlation analysis was performed using the nonparametric Spearman coefficient. A two-tailed p value ≤ 0.05 was considered statistically significant

The RHI values correlated negatively with age in the HC group (r = − 0.5405; p = 0.0375), as displayed in Fig. 2. In contrast, in patients with PCS, a positive correlation was found between RHI values and age (r = 0.5328; p = 0.0356). Furthermore, the RHI was positively correlated with systolic blood pressure (r = 0.6490; p: 0.0078), diastolic blood pressure (r = 0.5283; p = 0.0374) and BMI (r = 0.5843; p = 0.0193) in PCS patients. None of these associations were found in the ME/CFS patient group. In addition, no associations of the RHI with the Chalder Fatigue Scale, Bell Disability Scale or PEM score were observed (Additional file 1: Table S1).

### Alterations in endothelial biomarkers in post-COVID cohorts

**Fig. 3:**
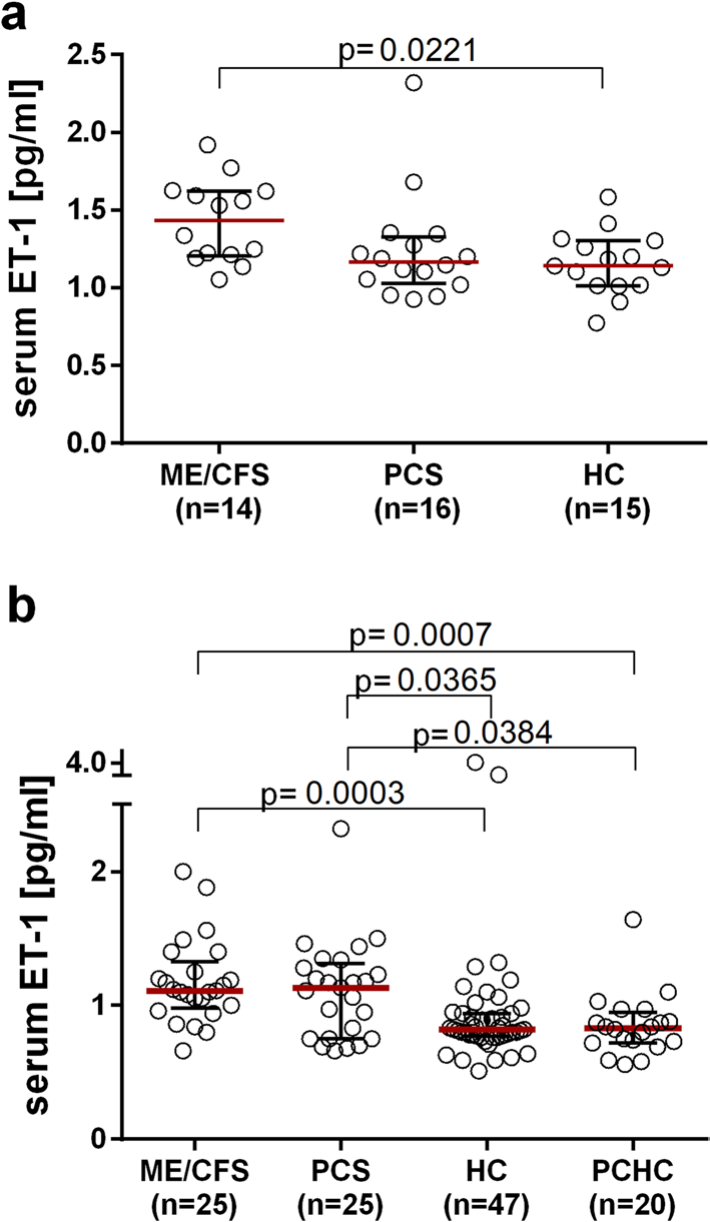
Serum ET-1 concentrations. The serum ET-1 concentrations were measured in the PAT study cohort (**a**) and in a second validation cohort (**b**). The median (IQR) serum ET-1 concentration is shown. For statistical analysis, the Kruskal–Wallis with Dunn´s post-hoc multiple comparisons test was used. P values ≤ 0.05 were considered statistically significant. *ET-1* Endothelin-1; *IQR* interquartile range; *RH-PAT* reactive hyperaemia peripheral arterial tonometry

The endothelial biomarkers ET-1, Ang-2 and ESM-1 were comparatively analysed in patient and control serum by ELISA in the RH-PAT study cohort. ET-1 concentrations in ME/CFS patients were significantly higher than those in HCs analysed in the same assay (p = 0.0221, Fig. 3A, Table 2A). In the validation cohort, significantly increased levels of ET-1 were confirmed for ME/CFS patients compared to HCs (p = 0.0003, Fig. 3B, Table 2B). Additionally, ET-1 levels were significantly higher than those in PCHCs (p = 0.0007, Fig. 3B, Table 2B). In PCS patients, the ET-1 serum concentration was also higher than that in both HCs (p = 0.0365, Fig. 3B, Table 2B) and PCHCs (p = 0.0384, Fig. 3B, Table 2B). The median duration post-COVID-19 in patients and PCHCs was eight and six months, respectively (Table 1B).

**Table 2:** Levels of biomarkers

(A) PAT study group	ME/CFS		PCS		HC		*p* value*
	Median (IQR)	n	Median (IQR)	n	Median (IQR)	n	
Serum ET-1 (pg/ml)	1.434 (1.208–1.622)	14	1.168 (1.019–1.328)	16	1.141 (1.014–1.302)	15	p_1_ = 0.0804 **p**_**2**_** = 0.0221** p_3_ > 0.9999
Serum Esm-1 (ng/ml)	2.013 (1.199–4.890)	14	2.013 (1.366–6.223)	16	1.782 (0.984–3.399)	15	p_1_ > 0.9999 p_2_ > 0.9999 p_3_ > 0.9999
Serum Ang-2 (pg/ml)	1866 (1502–2108)	14	1621 (1283–1762)	16	2058 (1566–3459)	15	p_1_ = 0.9341 p_2_ = 0.4715 **p**_**3**_** = 0.0379**
Serum ACE (U/l)	25.20 (17.70–32.35)	14	27.50 (23.65–32.65)	16	Normal range**: 20–70		p_1_ = 0.4043
Serum ACE2 (U/ml)	5.099 (4.192–6.162)	14	4.476 (3.946–6.476)	16	4.971 (4.23–6.792)	15	p_1_ > 0.9999 p_2_ > 0.9999 p_3_ = 0.5715
IL-8 post-erythrocyte lysis (pg/ml)	139.9 (116.7–197.6)	14	188.2 (150.2–217.0)	16	Normal range**: < 150		p_1_ = 0.0592
	> 150	7	> 150	12			

(B) Validation study group	ME/CFS		PCS		HC		PCHC		*p* value*
	Median (IQR)	n	Median (IQR)	n	Median (IQR)	n	Median (IQR)	n	
Serum ET-1 (pg/ml)	1.110 (0.980–1.325)	25	1.130 (0.750–1.310)	25	0.820 (0.770–0.940)	47	0.830 (0.723–0.948)	20	p_1_ > 0.9999 **p**_**2**_** = 0.0003** **p**_**3**_** = 0.0384** **p**_**4**_** = 0.0007** **p**_**5**_** = 0.0365** p_6_ > 0.9999
Serum Ang-2 (pg/ml)	2759 (2193–3425)	26	2149 (1564–2535)	30	2977 (2355–4143)	50	2356 (1802–3027)	20	**p**_**1**_** = 0.0188** p_2_ > 0.9999 **p**_**3**_** < 0.0001** p_4_ = 0.5336 p_5_ > 0.9999 **p**_**6**_** = 0.0234**
Serum ACE (U/l)	29.20 (20.10–42.20)	23	23.70 (19.43–31.75)	30	Normal range**: 20–70		NA		p_1_ = 0.1643
IL-8 post-erythrocyte lysis (pg/ml)	172.6 (136.9–198.0)	25	150.2 (126.7–180.1)	29	Normal range**: < 150		NA		p_1_ = 0.0884
	> 150	17	> 150	15					

*For comparative analysis of more than two groups, the Kruskal–Wallis test with Dunn´s post-hoc multiple comparisons was used, otherwise the Mann–Whitney-U rank-sum-test was used. A p value ≤ 0.05 was considered statistically significant. p_1_: ME/CFS vs. PCS, p_2_: ME/CFS vs. HC; p_3_: PCS vs. HC, p_4_: ME/CFS vs. PCHC, p_5_: PCS vs. PCHC, p_6_: HC vs. PCHC

**Normal range as stated by Labor Berlin

*ACE*Angiotensin-converting enzyme, *Ang-2*Angiopoietin-2, *Esm-1* Endocan, *ET-1* Endothelin-1, *HC* healthy control, *IQR*=interquartile range, *NA* not assessed

**Fig. 4:**
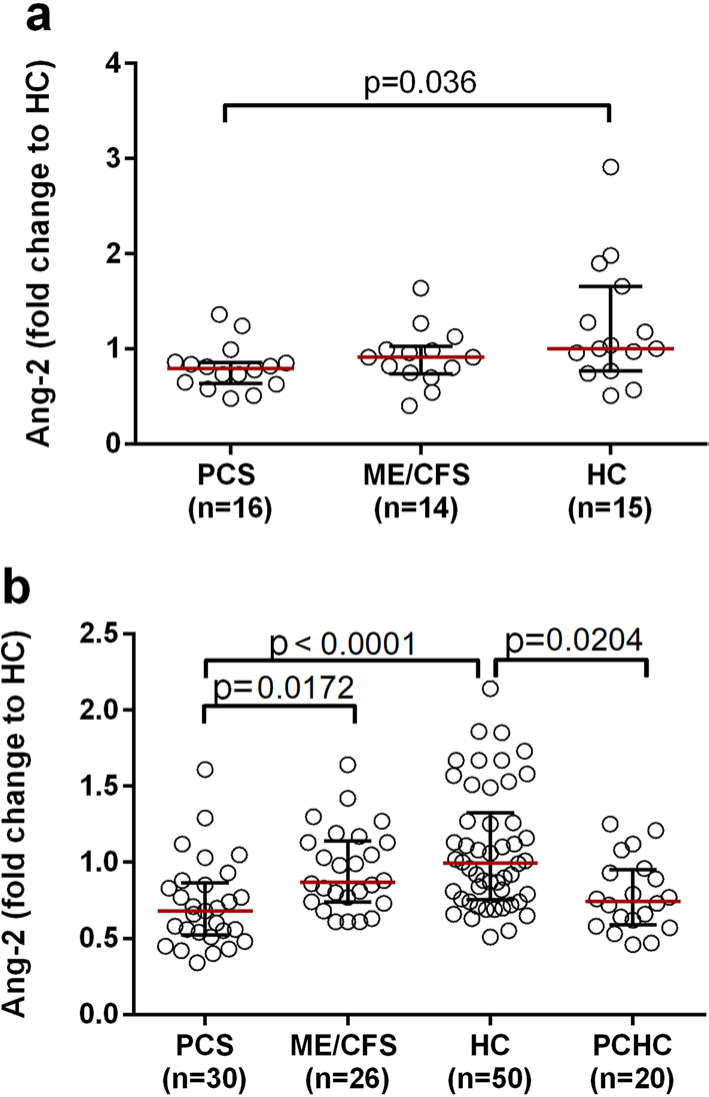
Serum Ang-2 levels. Serum Ang-2 levels were measured in the PAT study cohort (**a**) and in a second validation cohort (**b**). Levels are depicted as the median (IQR) of the fold change (FC) compared to that in HCs, as the standard was 1.5-fold higher in the ELISA of the ^nd^ validation cohort compared to the PAT study cohort. For statistical analysis, the Kruskal–Wallis with Dunn´s post-hoc multiple comparisons test was used. P values ≤ 0.05 were considered statistically significant. [Ang-2 = Angiopoietin-2; IQR = interquartile range; RH-PAT = reactive hyperaemia peripheral arterial tonometry]

In the RH-PAT study cohort, Ang-2 levels were significantly lower in PCS patients compared to HCs (P = 0.036, Fig. 4A). Significantly decreased Ang-2 levels in PCS patients compared to HCs were confirmed in the validation cohort (p < 0.0001, Fig. 4B, Table 2B). Furthermore, the Ang-2 levels were lower in PCS patients than in post- COVID ME/CFS patients (p = 0.0172, Fig. 4B, Table 2B). In addition, the Ang-2 levels were also lower in PCHCs compared to HCs (p = 0.0204, Fig. 4B, Table 2B). No significant difference in ESM-1 concentration between the cohorts was observed (Table 2A).

Serum ACE and IL-8 (measured following erythrocyte lysis) were determined in the diagnostic laboratory in patients and soluble ACE2 was analysed by ELISA (Table 2). The median levels of ACE did not differ between the two cohorts, but were below the normal reference value in five of the 14 ME/CFS patients and in two of the 16 PCS patients (< 20 U/l) as well as in eight of the 23 ME/CFS patients and five of the 30 PCS patients of the validation cohort. ACE2 levels did not differ between the two patient cohorts. The median IL-8 concentrations did not differ between patient cohorts but were above the normal reference value (> 150 pg/ml) in 12 of the 16 PCS patients and seven of the 14 ME/CFS patients in the PAT cohort as well as in 17 of the 25 ME/CFS patients and 15 of the 29 PCS patients in the validation cohort.

Neither the ET-1, Ang-2 or ESM-1 concentrations nor the ACE, ACE2 and IL-8 levels correlated with the RHI (Table 3).

**Table 3:** Correlation of the RHI with soluble biomarkers

	RHI	
	r, p value*	n
**ME/CFS**		
ET-1 (pg/ml)	− 0.108, p = 0.716	14
Ang-2 (pg/ml)	− 0.174, p = 0.553	14
ACE activity (U/l)	− 0.083, p = 0.788	13
ACE2 (U/ml)	0.503, p = 0.069	14
IL-8 post-erythrocyte lysis (pg/ml)	− 0.130, p = 0.659	14
**PCS**		
ET-1 (pg/ml)	0.162, p = 0.549	16
Ang-2 (pg/ml)	− 0.165, p = 0.541	16
ACE activity (U/l)	− 0.041, p = 0.882	16
ACE2 (U/ml)	− 0.359, p = 0.173	16
IL-8 post-erythrocyte lysis (pg/ml)	− 0.057, p = 0.833	16
**HC**		
ET-1 (pg/ml)	− 0.179, p = 0.524	15
Ang-2 (pg/ml)	0.125, p = 0.657	15
ACE2 (U/ml)	0.386, p = 0.157	15

*For statistical analysis, the Spearman correlation was performed

*ACE* Angiotensin-converting enzyme, *Ang-2*Angiopoietin-2; *ET-1* Endothelin-1, *HC* healthy control; *RHI* reactive hyperaemia index

## Discussion

Long COVID is a poorly understood condition with multiple features and a broad range of symptoms. Endothelial infection in COVID-19 may have long-term consequences for vascular function. In our present study, we found ED and dysregulated levels of the endothelial markers ET-1 and Ang-2 in a subset of PCS patients eight months, on average, after mild to moderate COVID-19.

In acute COVID-19, there is ample evidence for ED and impaired microcirculation [16, 34]. SARS-CoV-2 alters vascular homeostasis by directly infecting endothelial cells via ACE2 [16]. ACE2 is expressed in arterial and venous endothelial cells [35] and is internalized and downregulated after binding of the virus. The local angiotensin II hyperreactivity that is triggered by this action leads to the progression of inflammation and fibrosis [36]. In addition to direct injury to the vascular endothelium by endothelial cell infection, inflammatory mediators can also contribute to endotheliitis and endothelial cell injury [37]. There is also evidence for endothelial damage, especially in pulmonary microvascular cells, by apoptosis or autophagy in postacute COVID-19 [16, 38].

Furthermore, there is evidence of ED occurring in patients following infection with SARS-CoV-2. A recent study analysed endothelial function using EndoPAT technology in patients during the acute infection as well as a median of 100 days post-COVID-19; the study reported impaired RHI in the postinfection stage only [39]. However, in this study, no information about the severity of acute COVID-19 or symptom persistence was reported. Another study of patients after severe acute COVID-19 found impaired ED in half of them, which was associated with fatigue, chest pain, neurocognitive difficulties and severity of the acute COVID-19 [40]. The patients included in our study all had mild to moderate COVID-19. Thus, from our rather homogeneous patient cohort, we cannot draw conclusions regarding the impact of acute COVID-19 severity on endothelial function. A recent study reported elevated levels of circulating endothelial cells in COVID-19 convalescents on average 34 days post-symptom onset as a biomarker for ED associated with levels of several cytokines [41].

ED has been described in non-COVID-19 postinfectious ME/CFS [9–12]. In our previous study of 35 ME/CFS patients, peripheral ED assessed by RH-PAT was observed in half of the patients and was associated with disease severity [9]. A recent study of postinfectious ME/CFS confirmed these findings by demonstrating ED through both RH assessment and flow-mediated dilatation [11]. ED resulting in muscle and cerebral hypoperfusion are considered key pathomechanisms in ME/CFS [7, 12, 42].

The RHI in healthy individuals is usually inversely associated with age, blood pressure, BMI and further known cardiovascular risk factors [43], which we also observed for RHI and age in our HC group. Surprisingly, we found a paradoxical positive correlation of the RHI with age, blood pressure and BMI in PCS which may suggest that ED develops independently of classical cardiovascular risk factors in these patients and that vascular stiffness may even help to stabilise the vascular diameter. Further comparative studies of patients with PCS and of post-COVID reconvalescents are required to provide evidence whether a diminished RHI might be associated with PCS.

Remarkably, PCS patients with and without ME/CFS showed elevated levels of the endothelial biomarker ET-1, while reconvalescents had normal levels. Endothelins are the most important, potent vasoconstrictors and are produced by endothelial cells [18, 19]. ET-1 mediates vasoconstriction via the ETA receptor, which is mainly located on vascular smooth muscle cells. Thus, our findings may indicate hypoperfusion in PCS, which is in line with a recent study in PCS patients with exertion intolerance. Data from cardiopulmonary exercise testing (CPET) provide evidence for a marked reduction in oxygen consumption during exercise, which is attributed primarily to reduced oxygen diffusion in the peripheral microcirculation [44]. Targeting the ETA and ETB receptors via an antagonist improved the peripheral endothelial function defined by RHI in patients with type 2 diabetes [45]. Thus, ET-1 may be both a biomarker of endothelial involvement and a therapeutic target in PCS.

Ang-2 belongs to the angiopoietin/tie-2 pathway that regulates endothelial homeostasis and angiogenesis. In the present study, non-ME/CFS PCS patients unexpectedly showed reduced serum Ang-2 levels compared to those in HCs. Ang-2 expression is increased during COVID-19 infection presumably due to endothelial inflammation [20]. Endothelial cells were shown to downregulate Ang-2 expression under high flow and shear stress [46]. Thus, a possible explanation may be the occurrence of high shear stress in PCS due to chronic inflammation or endothelial damage [47]. Remarkably, Ang-2 was also diminished in reconvalescent PCHC, which may also indicate a longer lasting change in vascular perfusion in asymptomatic individuals. With increased levels of ET-1 in both patient groups, the finding of decreased Ang-2 levels exclusively in PCS could provide a starting point for differentiation between PCS and ME/CFS in terms of biomarker profiles.

As reported in our previous study [4], we found elevated levels of the pro-inflammatory mediator IL-8 in approximately 60% of PCS patients. As IL-8 is rapidly degraded in serum, its concentration was determined in lysed erythrocytes, which bind IL-8 via a Duffy antigen receptor [48, 49]. Endothelial cells and monocytes are the main producers of IL-8 [23, 49]. IL-8 promotes endothelial cell migration and proliferation as well as survival and endothelial permeability [23, 50]. Elevated IL-8 levels were described in patients with severe as well as mild COVID-19 and correlated with disease prognosis [51]. Therefore, IL-8 might indicate ongoing endothelial inflammation in our PCS patients.

## Conclusion

In conclusion, our study found that a subset of PCS patients had a diminished RHI, indicating peripheral ED. A limitation of our study is the lack of a reconvalescent cohort for RHI assessment; thus, we do not know whether a diminished RHI is associated with PCS symptoms. Elevated ET-1 levels were, however, found in PCS patients only, indicating that endothelial hypoperfusion plays a role in PCS and providing a rationale for therapeutic targeting. The paradoxical association of RHI with age, blood pressure and BMI as well as diminished Ang-2 may indicate a distinct pathomechanism in the non-ME/CFS PCS subgroup.

## Supplementary Information

**Additional file 1: Table S1.** Clinical parameters investigated for correlation with the reactive hyperemia index (RHI).

## Data Availability

The datasets used and/or analysed during the current study are available from the corresponding author on reasonable request.

## Abbreviations

ACE: Angiotensin-converting enzyme
Ang: Angiopoietin
ED: Endothelial dysfunction
Esm-1: Endocan
ETA/B receptor: Endothelin receptor type A/B
ET-1: Endothelin-1
HC: Healthy control
IQR: Interquartile range
ME/CFS: Myalgic encephalomyelitis/chronic fatigue syndrome
NA: Not assessed
PCHC: Post-COVID healthy control
PCS: Post-COVID syndrome
RHI: Reactive hyperaemia index
RH-PAT: Reactive hyperaemia peripheral arterial tonometry
