# Supplementary material for: Endothelial dysfunction and altered endothelial biomarkers in patients with post-COVID-19 syndrome and chronic fatigue syndrome (ME/CFS)
Source: J Transl Med. 2022 Mar 22;20:138. doi: 10.1186/s12967-022-03346-2 (PMC8938726; doi:10.1186/s12967-022-03346-2)
Supplement: Supplementary file 1 — Additional file 1: Table S1. Clinical parameters investigated for correlation with the reactive hyperemia index (RHI). [file 12967_2022_3346_MOESM1_ESM.docx]

Supplementary data

Table S1: Clinical parameters investigated for correlation with the reactive hyperemia index (RHI)

| **Parameter** | **ME/CFS** | | **PCS** | |
| --- | --- | --- | --- | --- |
|  | **n** | **Spearman’s r,**  **p value** | **n** | **Spearman’s r,**  **p value** |
| Total DSQ-PEM Score | 14 | r: -0.1940 p: 0.5033 | 16 | r: -0.081 p: 0.764 |
| Total Chalder Fatigue Scale | 14 | r: 0.2212 p: 0.4442 | 16 | r: -0.076 p: 0.780 |
| Bell Disability Scale | 14 | r: 0.3210 p: 0.2619 | 16 | r: -0.398 p: 0.127 |

Spearman correlation was performed. A p value ≤ 0.05 was considered statistically significant.

[PEM = post exertional malaise]
